# Inhibiting the immunoproteasome exacerbates the pathogenesis of systemic *Candida albicans* infection in mice

**DOI:** 10.1038/srep19434

**Published:** 2016-01-18

**Authors:** Sarah Mundt, Michael Basler, Stefanie Buerger, Harald Engler, Marcus Groettrup

**Affiliations:** 1 Division of Immunology, Department of Biology, University of Konstanz, Konstanz, Germany; 2 Konstanz Research School Chemical Biology (KoRS-CB), University of Konstanz, Konstanz, Germany; 3 Biotechnology Institute Thurgau (BITg) at the University of Konstanz, Kreuzlingen, Switzerland; 4 FlowKon Core Facility for Cell Sorting and Flow Cytometry, University of Konstanz, Konstanz, Germany; 5 Institute of Medical Psychology and Behavioural Immunobiology, University Hospital Essen, University of Duisburg-Essen, Essen, Germany

## Abstract

Apart from its role in MHC class I antigen processing, the immunoproteasome has recently been implicated in the modulation of T helper cell differentiation under polarizing conditions *in vitro* and in the pathogenesis of autoimmune diseases *in vivo*. In this study, we investigated the influence of LMP7 on T helper cell differentiation in response to the fungus *Candida albicans*. We observed a strong effect of ONX 0914, an LMP7-selective inhibitor of the immunoproteasome, on IFN-γ and IL-17A production by murine splenocytes and human peripheral blood mononuclear cells (PBMCs) stimulated with *C. albicans in vitro*. Using a murine model of systemic candidiasis, we could confirm reduced generation of IFN-γ- and IL-17A-producing cells in ONX 0914 treated mice *in vivo*. Interestingly, ONX 0914 treatment resulted in increased susceptibility to systemic candidiasis, which manifested at very early stages of infection. Mice treated with ONX 0914 showed markedly increased kidney and brain fungal burden which resulted in enhanced neutrophil recruitment and immunopathology. Together, these results strongly suggest a role of the immunoproteasome in promoting proinflammatory T helper cells in response to *C. albicans* but also in affecting the innate antifungal immunity in a T helper cell-independent manner.

The 26S proteasome is a multicatalytic enzyme expressed in the nucleus and cytoplasm of all eukaryotic cells. It is responsible for the degradation of the bulk (80–90%) of cellular proteins thereby regulating many biological processes including major histocompatibility complex (MHC) class I antigen presentation^1^,^2^,^3^. In response to the proinflammatory cytokine interferon (IFN)-γ, the catalytically active β subunits of the proteasome are replaced by their inducible counterparts low molecular mass polypeptide (LMP)2 (β1i), multicatalytic endopeptidase complex-like (MECL)-1 (β2i), and LMP7 (β5i), building the so-called immunoproteasome^4^,^5^. The incorporation of the inducible subunits leads to minor structural changes within the proteasome, to a marked change in the cleavage site preference, and to an enhanced production of T cell epitopes^4^,^6^. However, it has been demonstrated that immunoproteasomes do not only play an important role in MHC class I antigen processing but also in shaping the naive T cell repertoire in the thymus and regulating immune responses in the periphery^7^,^8^. ONX 0914 (formerly named PR-957), an LMP7-selective epoxyketone inhibitor of the immunoproteasome, reduced cytokine production in activated monocytes or lymphocytes *in vitro* and attenuated disease progression in various mouse models of autoimmune diseases^7^,^9^,^10^,^11^,^12^,^13^,^14^. Moreover, selective inhibition of LMP7 was shown to suppress the development of T helper (Th) 1 and Th17 cells and to promote regulatory T (Treg) cell generation under polarizing conditions *in vitro*^9^,^11^

*Candida albicans* is a commensal organism of mucosal and skin surfaces which can cause various disease manifestations ranging from oral or mucocutaneous to lethal disseminated candidiasis in immunocompromised hosts^15^,^16^. Host protection from infection ultimately depends on the recognition of *C. albicans* by pattern recognition receptors (PRRs) and their associated signalling pathways that initiate antifungal immunity. *C. albicans* is a strong inducer of Th1 and Th17 cell differentiation by the engagement of C-type lectins on the surface of antigen presenting cells and the subsequent induction of cytokines like interleukin (IL)-6, IL-12, IFN-γ, and IL-23^17^,^18^,^19^,^20^. Th1 cells provide fungal control through IFN-γ production required for optimal activation of phagocytes and for helping in the generation of a protective antibody response^21^. Th17 cells act as an important source of IL-17A which is crucial for the anti-*C. albicans* host defence by inducing the expression of genes encoding proinflammatory cytokines, chemokines, and antimicrobial peptides, as well as by promoting granulopoiesis and recruiting neutrophils to the site of infection^19^,^22^,^23^. Despite adaptive immunity being important for host defence against mucocutaneous candidiasis, it does not play a prominent role in combatting disseminated *C. albicans* infection. Instead, innate immunity acts as the major barrier to systemic *Candida* spread. The candidacidal activity of neutrophils is the key mediator of immunity to systemic candidiasis and neutropenia is a major risk factor for invasive *candidiasis*^24^,^25^. As in humans, the mouse kidney is the primary target organ during systemic *C. albicans* infection and progressive sepsis as well as renal failure account for mortality in that model^26^,^27^,^28^. Since the severity of kidney damage is quantitatively related to the levels of host innate immune responses, it has been suggested that uncontrolled inflammation and subsequent immunopathology, rather than *C. albicans* itself, may worsen disease outcome. Indeed, massive infiltration of neutrophils is commonly observed and believed to contribute significantly to host tissue destruction^29^,^30^,^31^.

Here, we found that ONX 0914 treatment blocks Th1 and Th17 cell differentiation in response to *C. albicans in vitro* and in a murine model of systemic candidiasis *in vivo*. Interestingly, ONX 0914 treated mice displayed an enhanced susceptibility at early stages of infection implicating a so far undescribed influence of LMP7 inhibition on innate anti-*C. albicans* immune responses.

## Results

### Reduced *C. albicans*-induced production of IL-17A and IFN-γ by murine splenocytes and human PBMCs *in vitro*

**Figure 1:**
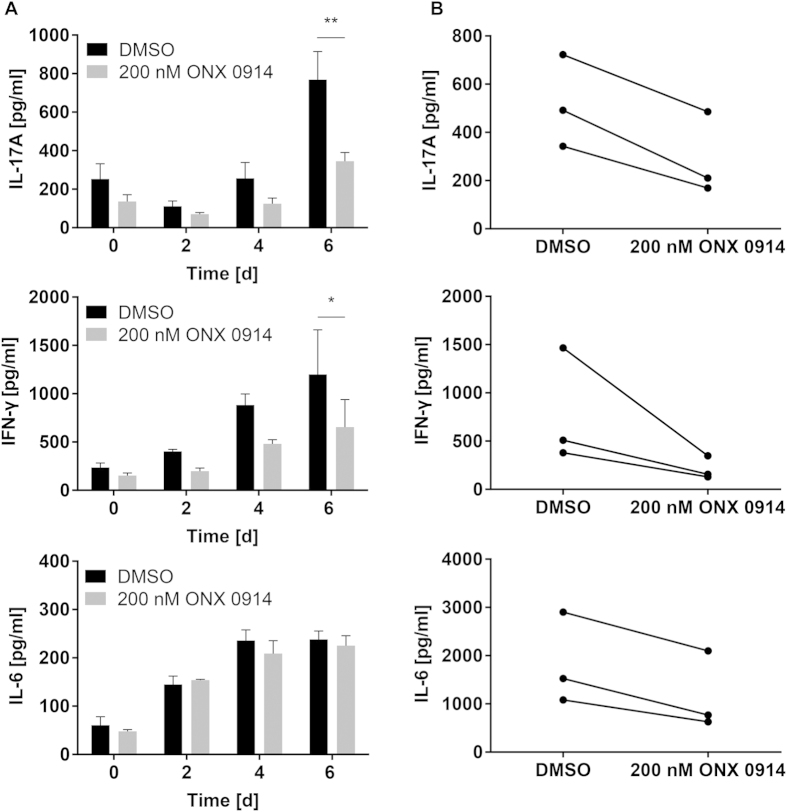
Influence of ONX 0914 on *C. albicans*-induced production of IL-6, IL-17A, and IFN-γ by murine splenocytes and human PBMCs *in vitro*. Culture supernatant levels of IL-6, IL-17A, and IFN-γ were measured by ELISA. (**A**) Naive murine splenocytes were pulsed with DMSO or 200 nM ONX 0914 for 2 h and cultured in the presence of heat-killed *C. albicans* yeast for up to 6 days. Data are representative for one out of three independent experiments and expressed as mean +/− SD. (**B**) Human PBMCs from healthy donors were treated as described in (**A**) and cultured in the presence of heat-killed *C. albicans* hyphae for 5 days. Data represent blood samples from three different donors. Data are analyzed by two-way ANOVA with *p < 0.05 and **p < 0.01.

Hitherto, the inhibition of LMP7 has been shown to suppress several autoimmune diseases which correlated with a reduction in the differentiation of pathogenic Th1 and Th17 cells. Since Th1 and especially Th17 cells play a central role in the host defence against *C. albicans*, we investigated the impact of immunoproteasome inhibition on the immune response against this pathogen. In a first approach, we stimulated naive splenocytes with heat-killed *C. albicans* yeast *in vitro*, leading to IL-17A and IFN-γ release into the culture supernatant. Compared to cells treated with DMSO, we observed reduced IL-17A and IFN-γ production by splenocytes pulsed with 200 nM ONX 0914 (Fig. 1A). This effect was LMP7-dependent because ONX 0914 treatment of splenocytes from LMP7^−/−^ mice did not lead to reduced cytokine production (Supplementary Fig. S1). In order to investigate whether LMP7 inhibition has a similar effect on human peripheral blood mononuclear cells (PBMCs), PBMCs from healthy volunteers were stimulated with heat-killed *C. albicans in vitro* and cytokines were measured in the supernatant by ELISA. Similar to murine splenocytes, ONX 0914 treatment of human PBMCs resulted in reduced IL-17A and IFN-γ production in response to *C. albicans* (Fig. 1B). Interestingly, the secretion of IL-6, a key cytokine for Th17 differentiation, was reduced in human PBMCs by LMP7 inhibition while it was not affected in murine splenocytes (Fig. 1A). Next, naive murine splenocytes were negatively sorted for CD4^+^ T cells and either the ‘antigen presenting cells’-containing fraction (splenocytes - CD4^+^ T cells) or the CD4^+^ T cell fraction was pulsed with 200 nM ONX 0914. Untreated and treated cell fractions were combined and stimulated with heat-killed *C. albicans*. IFN-γ and IL-17A production was also strongly reduced by ONX 0914 treatment of only the ‘antigen presenting cells’-containing fraction (splenocytes - CD4^+^ T cells) (Supplementary Fig. S2A). Similarly, the release of IFN-γ and IL-17A was reduced by treatment of the CD4^+^ T cell fraction, although not quite significant for IFN-γ (Supplementary Fig. S2B). Moreover, when splenocytes were stimulated with *C. albicans* in the presence of blocking antibodies to MHC-II, we observed a strong reduction of IL-17A (Supplementary Fig. S2C) indicating that IL-17A production is to a large degree dependent on MHC-II antigen presentation. These results indicate that ONX 0914 is able to block the production of proinflammatory cytokines by directly acting on CD4^+^ T cells as well as affecting ‘antigen presenting cells’.

### Impaired generation of IL-17A- and IFN-γ- producing cells and aggravated clinical outcome of disseminated candidiasis in ONX 0914 treated mice

**Figure 2:**
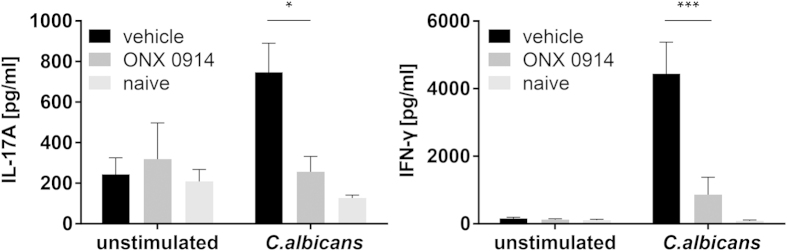
LMP7 inhibition reduces the development of IL-17A and IFN-γ producing cells *in vivo*. Mice were i.v. infected with 1 × 10^5^ CFU live *C. albicans* blastoconidia and treated with 10 mg/kg ONX 0914 (s.c.) every second day. On day 7 postinfection, splenocytes were restimulated with 10^6^/ml heat-killed *C. albicans* yeast cells for 48 h. Levels of IL-17A (left graph) and IFN-γ (right graph) were measured in the supernatant by ELISA. Data are representative for one out of three independent experiments and expressed as mean +/− SEM of n = 5 mice (n = 2 naive mice). Data are analyzed by two-way ANOVA with *p < 0.05 and ***p < 0.001.

**Figure 3:**
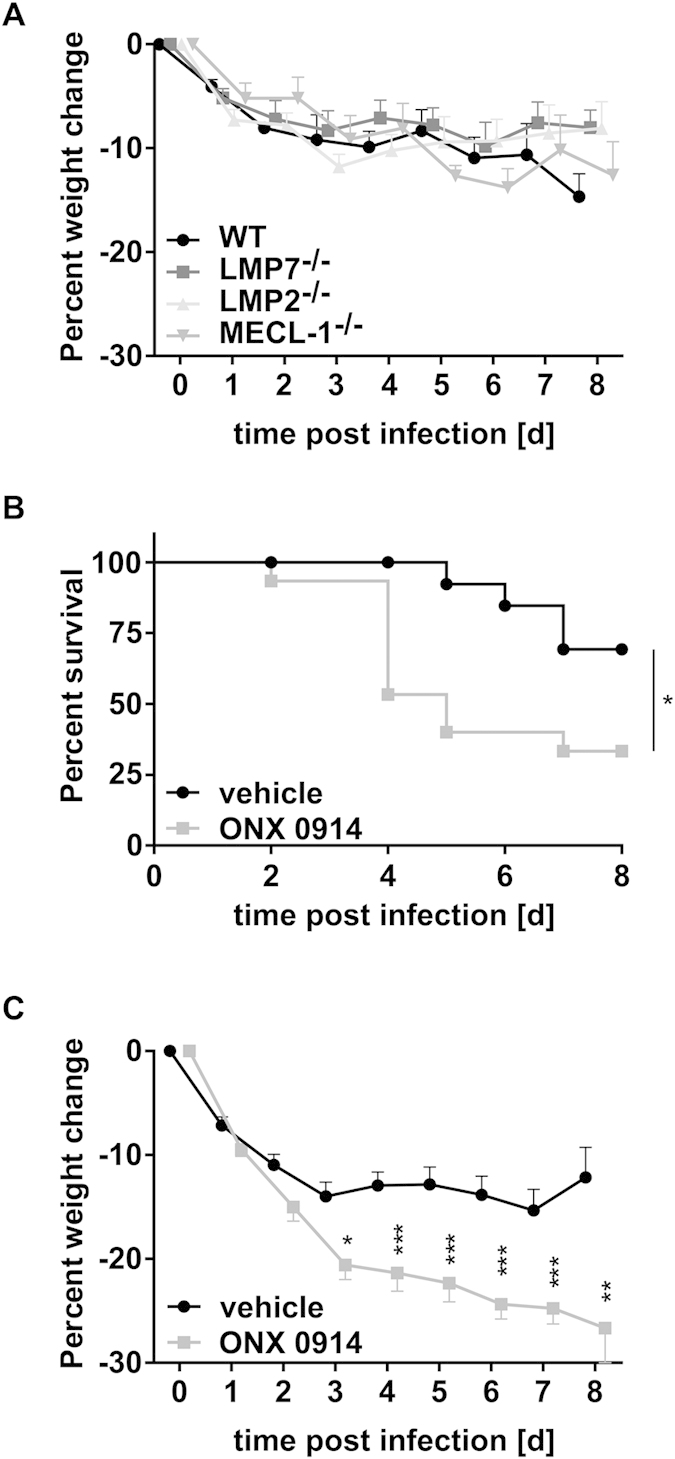
Influence of the immunoproteasome on weight loss and survival in systemic candidiasis. C57BL/6, LMP7^−/−^, LMP2^−/−^, and MECL-1^−/−^ mice were i.v. infected with 1 × 10^5^ CFU live *C. albicans* blastoconidia and treated with vehicle or 10 mg/kg ONX 0914 (s.c.) every second day where indicated (**B**,**C**). (**A**,**C**) Change of body weight during systemic infection with *C. albicans*. Percent weight loss (y-axis) is plotted versus time (x-axis). Data points represent mean weight change +/− SEM. (**B**) Survival curves of ONX 0914 and vehicle treated mice. Data represent combined results from 2–3 experiments with n = 8–18 mice per group. Data are analyzed by two-way ANOVA with *p < 0.05, **p < 0.01, and ***p < 0.001.

Our results obtained with splenocytes and PBMCs stimulated with *C. albicans in vitro* (Fig. 1) and previous reports suggest a role of LMP7 inhibition in T helper cell differentiation^9^,^11^,^13^. Thus, we intended to investigate whether the immunoproteasome has an influence on the generation or activation of IL-17A- and IFN-γ-producing cells in response to systemic *C. albicans* infection *in vivo*. Analyzing cytokine production of splenocytes upon *ex vivo* restimulation with heat-killed *C. albicans* on day 7 postinfection revealed a marked reduction of IL-17A and IFN-γ production upon LMP7 inhibition *in vivo* (Fig. 2). While immunoproteasome-deficient mice showed no altered susceptibility to invasive candidiasis (Fig. 3A), ONX 0914 treated mice suffered from accelerated and more pronounced weight loss as well as higher mortality compared to vehicle treated mice already on day 2 of infection (Fig. 3B,C). Moreover, in some experiments, we observed that LMP7 inhibition led to movement disorders and neurological abnormalities of mice as manifested by a slight tilting of the head and uncontrolled twisting/rotation when handled (data not shown).

### Selective inhibition of LMP7 leads to increased fungal burden at early time points in the course of invasive candidiasis

**Figure 4:**
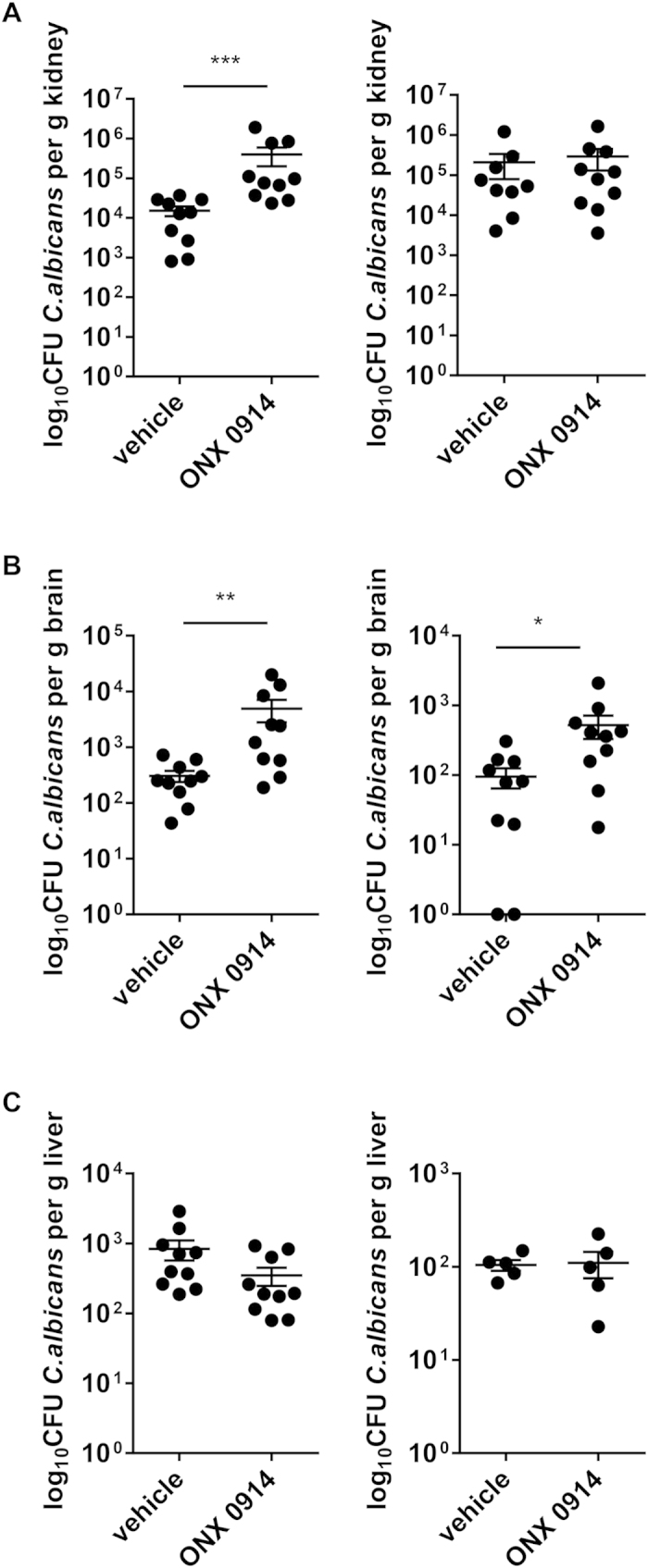
Influence of LMP7 inhibition on fungal burden in kidney, liver, and brain. Mice were intravenously infected with 1 × 10^5^ CFU live *C. albicans* blastoconidia and treated with vehicle or 10 mg/kg ONX 0914 (s.c.) every second day. On day 3 (left panels) and day 7 (right panels) fungal burden was determined in kidneys (**A**), brains (**B**), and livers (**C**). Bars show mean log_10_ CFU/g tissue +/− SEM. Data represent pooled results from two independent experiments and are analyzed by non-parametric Mann-Whitney test with *p < 0.05, **p < 0.01 and ***p < 0.001.

Mice with disseminated candidiasis die because of progressive sepsis and, notably, kidney fungal burden was shown to correlate with severity of renal failure and acidemia, which are hallmarks of severe sepsis^28^. Therefore, we investigated whether the observed enhanced susceptibility to systemic infection with *C. albicans* in ONX 0914 treated mice is due to defects in the control of the fungus. Kidney, brain, and liver tissue homogenates were analyzed for fungal outgrowth. Interestingly, mice treated with ONX 0914 had higher fungal burden in kidneys and brains compared to vehicle treated mice at early stages (day 3) of disseminated candidiasis (Fig. 4A,B). The increased fungal burden in the brain of ONX 0914 treated mice was also detectable at later stages (day 7) of infection (Fig. 4B). In contrast, in the liver (day 3 + 7) and at later time points in the kidney (day 7), LMP7 inhibition had no influence on the immune systems’ capability to control the fungus (Fig. 4A,C). Importantly, we found that ONX 0914 had no impact on the growth of *C. albicans* cultures *in vitro* (data not shown).

### Antifungal treatment is still effective in ONX 0914 treated mice

We wondered whether the increased susceptibility to systemic candidiasis observed upon LMP7 inhibition would be treatable with antifungal agents like Amphotericin B (AmpB). Mice receiving vehicle or ONX 0914 were daily treated with 10 mg/kg AmpB. Strikingly, these mice, no matter if treated with vehicle or ONX 0914, were almost fully protected from weight loss (Supplementary Fig. S3A). Analyzing renal fungal burden revealed that independent of LMP7 inhibition, AmpB treatment resulted in almost complete clearance of the fungus on day 7 postinfection (Supplementary Fig. S3B).

### Elevated neutrophil numbers in kidneys and brains of ONX 0914 treated mice during invasive candidiasis

**Figure 5:**
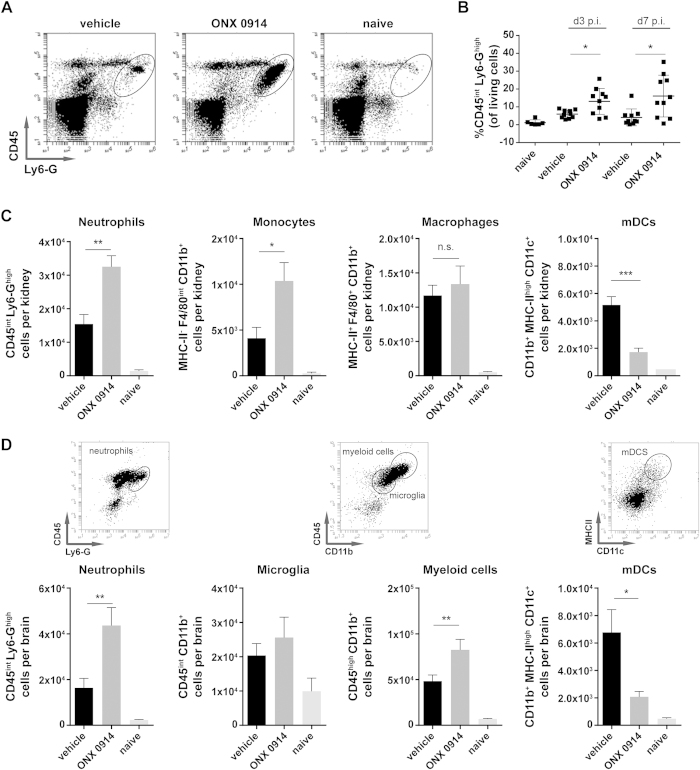
Elevated neutrophil numbers in the kidney and the brain of ONX 0914 treated mice. Mice were i.v. infected with 1.5 × 10^5^ CFU live *C. albicans* blastoconidia and treated with vehicle or 10 mg/kg ONX 0914 (s.c.) every second day. Kidneys and brains were removed and isolated leukocytes were stained for Ly6-G, CD45, F4/80, CD11b, MHC-II, and CD11c and analyzed by flow cytometry (gated on living cells according to FSC/SSC). Graphs show (**A**) representative flow cytometry profiles of kidney infiltrating CD45^int^Ly6-G^high^ neutrophils on day 7, (**B**) pooled results from two independent experiments presented as mean percentage of kidney infiltrating CD45^int^Ly6-G^high^ neutrophils +/− SEM, (**C**,**D**) pooled results from two independent experiments presented as mean absolute numbers +/− SEM of (**C**) kidney infiltrating CD45^int^Ly6-G^high^ neutrophils, MHCII^-^F4/80^int^CD11b^+^ monocytes, MHCII^+^ F4/80^+^ CD11b^+^ macrophages, and CD11b^+^MHCII^high^CD11c^+^ myeloid derived dendritic cells (mDCs) 48 h p.i., and (**D**) brain infiltrating CD45^int^Ly6-G^high^ neutrophils, CD45^high^CD11b^+^ myeloid cells, CD11b^+^MHCII^high^CD11c^+^ mDCs, and CD45^int^CD11b^+^ CNS resident microglia 72 h p.i. Data were analyzed by students t test with *p < 0.05, **p < 0.01, and ***p < 0.001.

Defects in IL-17 immunity are often associated with impaired neutrophil recruitment and function^22^,^32^. Hence, we investigated the influence of ONX 0914 treatment on neutrophil recruitment to the kidney, which is the main target of systemic candidiasis^26^,^27^,^28^,^33^. Unexpectedly, we observed significantly increased CD45^int^ Ly6-G^high^ neutrophil numbers in the kidneys of ONX 0914 treated mice both early (day 2 + 3) and late (day 7) in the course of invasive candidiasis (Fig. 5A–C) as detected by flow cytometric analysis of purified renal leukocytes. Moreover, we observed significantly increased numbers of kidney infiltrating F4/80^+^ CD11b^+^ MHC-II^-^ monocytes upon LMP7 inhibition 48 h postinfection (Fig. 5C). While the infiltration of F4/80^+^ CD11b^+^ MHC-II^+^ macrophages was not affected by LMP7 inhibition, we found reduced numbers of CD11b^+^ MHC-II^high^ CD11c^+^ myeloid dendritic cells (mDCs) in the kidney of ONX 0914 treated mice (Fig. 5C). Interestingly, we noticed a strong inflammatory infiltration of innate immune cells into the brain on day 3 of systemic infection with *C. albicans* in both, ONX 0914 and vehicle treated mice (Fig. 5D). Nevertheless, compared to vehicle treated mice, ONX 0914 treatment resulted in a strong increase of CD45^int^ Ly6-G^high^ neutrophils and CD45^high^ CD11b^+^ myeloid cells while infiltrating CD11b^+^ MHC-II^high^ CD11c^+^ mDCs numbers were reduced (Fig. 5D). CNS resident CD45^int^ CD11b^+^ microglia, which were demonstrated to be the main cell type responding to *C. albicans* in the brain^33^, were also increased upon infection. However, we observed no difference between vehicle and ONX 0914 treated mice.

**Figure 6:**
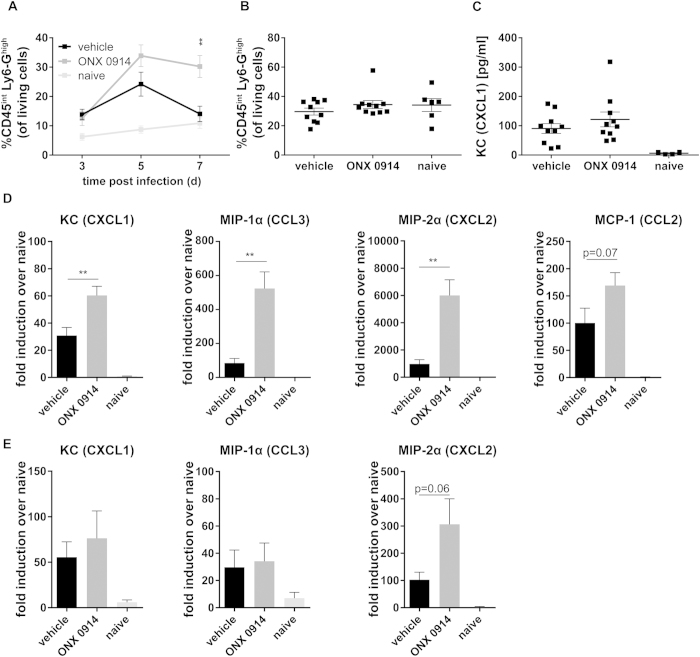
Influence of ONX 0914 treatment on neutrophil recruitment. Mice were i.v. infected with 1 × 10^5^ CFU live *C. albicans* blastoconidia and treated with vehicle or 10 mg/kg ONX 0914 (s.c.) every second day. (**A**) Peripheral blood (day 3, 5, and 7) and (**B**) bone marrow cells were stained for Ly6-G and CD45 and analyzed by flow cytometry. Graphs show mean percentage of CD45^int^Ly6-G^high^ neutrophils (gated on living cells according to FSC/SSC) +/− SEM. (**C**) Serum levels of KC (CXCL1) were determined by cytometric bead array. Graphs show pooled data from two independent experiments and are presented as mean +/− SEM. (**D**,**E**) Relative mRNA expression of KC (CXCL1), MIP-1α (CCL3), MCP-1 (CCL2), or MIP-2α (CCXL2) in the kidney on day 2 (**D**) or day 7 p.i. (**E**). Real-time RT-PCR data are pooled from two independent experiments with n = 8–10 mice per group and expressed as fold induction over naive +/− SEM relative to one out of four naive mice, which served as uninfected control. Data were analyzed by students t test with **p < 0.01.

In an attempt to identify the cellular source of the increase of neutrophilic granulocytes, we detected significantly elevated numbers of blood neutrophils in mice treated with ONX 0914, particularly at later stages of infection (Fig. 6A). However, when analyzing bone marrow neutrophils we could not find any difference in the percentage of mature CD45^int^ Ly6-G^high^ cells between vehicle and ONX 0914 treated mice on day 3 (Fig. 6B) or day 5 postinfection (data not shown) suggesting that the development of neutrophils in the bone marrow was not affected. In order to clarify the reason for the increased neutrophil numbers in blood and kidney upon LMP7 inhibition, we assessed the expression of important chemoattractants for monocytes and polymorphonuclear leukocytes. On day 3 postinfection, we detected increased keratinocyte-derived cytokine (KC) (CXCL1) serum cytokine levels (Fig. 6C) which were not altered by ONX 0914 treatment. In contrast, kidney mRNA levels of KC (CXCL1), macrophage inflammatory protein (MIP)-1α (CCL3), monocyte chemotactic protein (MCP)-1 (CCL2), and MIP-2α (CXCL2) were strongly upregulated upon LMP7 inhibition 48 h postinfection (Fig. 6D). At later stages (day 7) of systemic candidiasis, no difference was seen for KC and MIP-1α (CCL3), whereas MIP-2α (CXCL2) was still upregulated (Fig. 6E).

**Figure 7:**
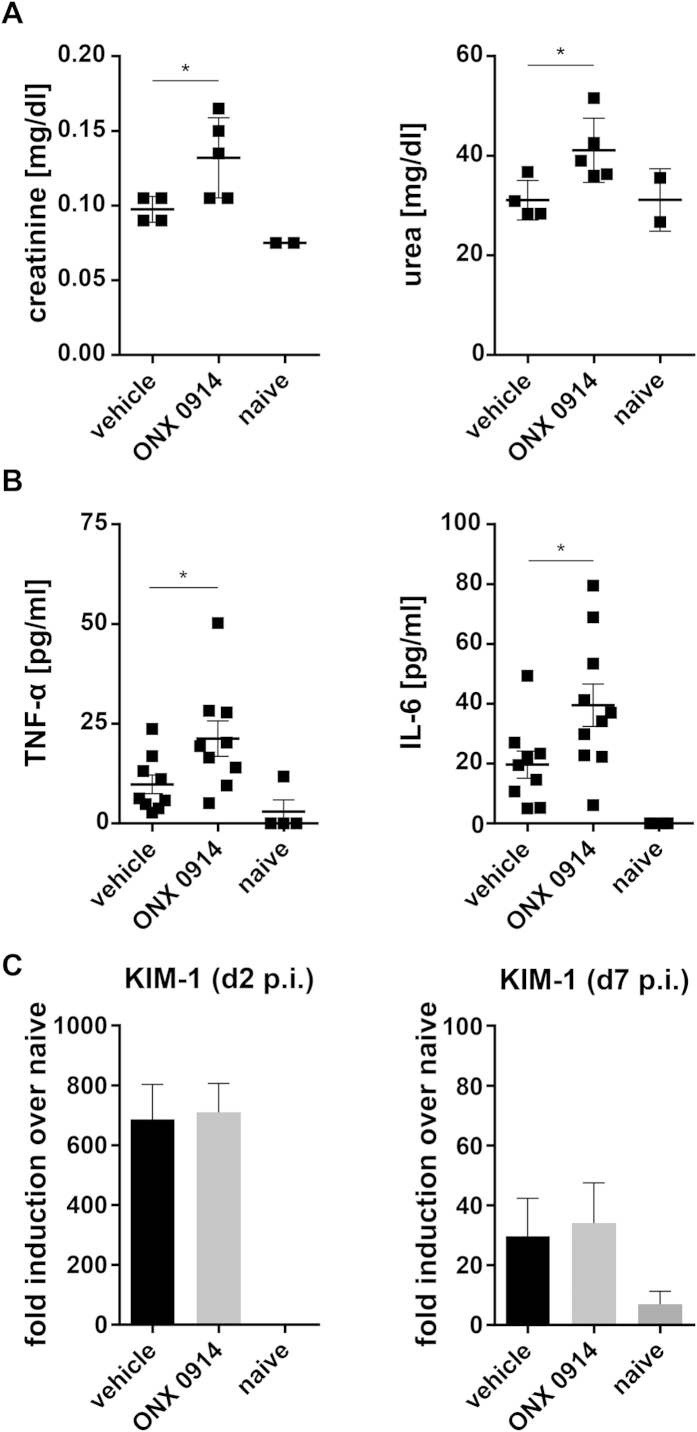
Influence of LMP7 inhibition on the maintenance of kidney function and proinflammatory serum cytokine levels. Mice were i.v. infected with 1 × 10^5^ CFU live *C. albicans* blastoconidia and treated with vehicle or 10 mg/kg ONX 0914 (s.c.) every second day. Graphs show (**A**) photometrically determined serum levels of urea and creatinine on day 7 and (**B**) serum levels of IL-6 and TNF-α as detected by cytometric bead array on day 3. (**C**) Relative mRNA expression of KIM-1 on day 2 and day 7, pooled from two independent experiments with n = 8–10 mice per group, and presented as fold induction over naive +/− SEM relative to one out of four naive mice, which served as uninfected control. Data are analyzed by students t test with *p < 0.05.

Enhanced neutrophil recruitment to the kidney during systemic candidiasis has been correlated with inflammation and immunopathology-mediated renal failure^31^. This would fit to the observation that ONX 0914 treated mice are more susceptible to invasive candidiasis and indicate that these mice might suffer from deregulated immunopathology leading to increased renal tissue damage. Indeed, in some cases, the kidneys of ONX 0914 treated mice appeared paler and more swollen than kidneys of vehicle treated mice by gross pathology (data not shown). In ONX 0914 treated mice we found elevated serum levels of creatinine and urea on day 7 (Fig. 7A) and of TNF-α and IL-6 on day 3 and day 7 (Fig. 7B; data not shown) indicating that renal failure and sepsis might occur faster upon LMP7 inhibition. Renal mRNA expression of kidney injury molecule-1 (KIM-1), a marker for early kidney damage, was strongly upregulated on day 2 and day 7 postinfection, but no difference could be detected between ONX 0914 and vehicle treated mice (Fig. 7C).

### Reduced activation of innate immune cells upon LMP7 inhibition *in vivo*

**Figure 8:**
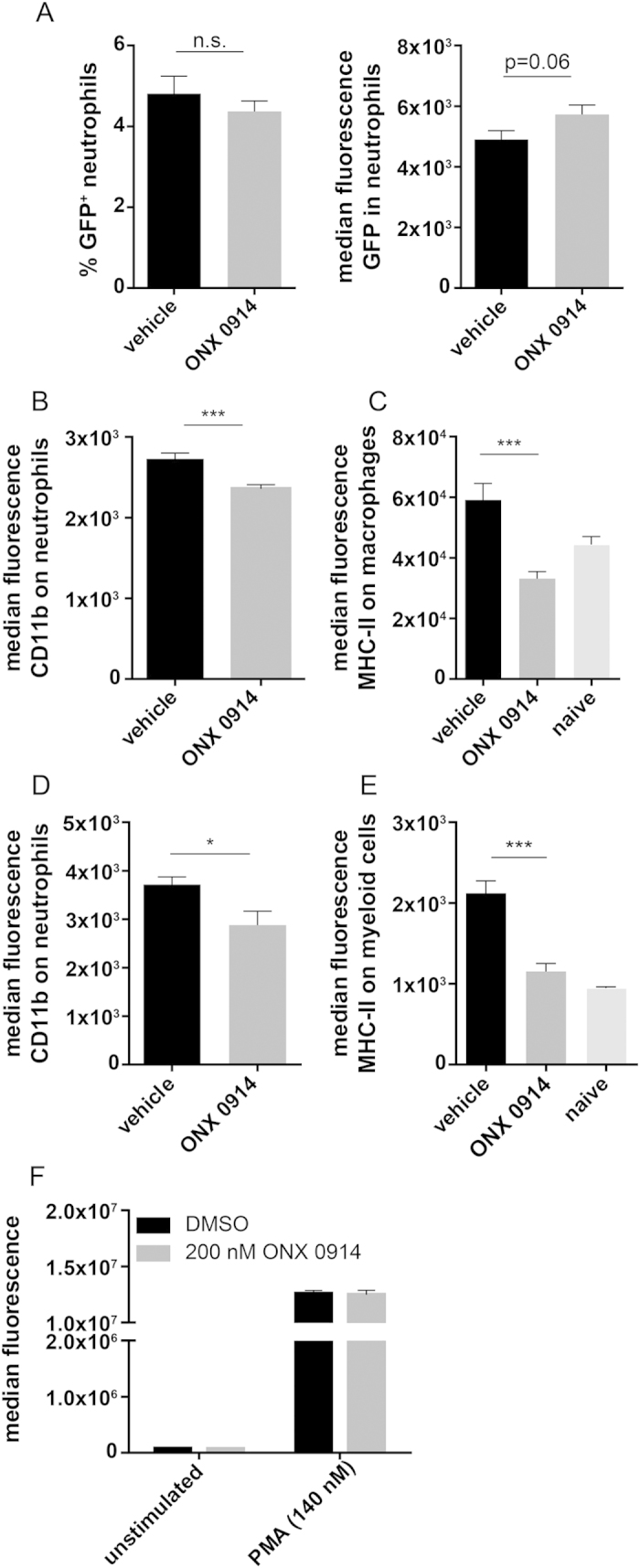
Influence of LMP7 inhibition on *C. albicans*-induced activation of innate immune cells. (**A**) Mice were intravenously infected with 1.5 × 10^5^ CFU live *C. albicans*-GFP blastoconidia and treated with vehicle or 10 mg/kg ONX 0914 (s.c.) on the day of infection. 48 h p.i., kidneys were removed and isolated leukocytes were stained for Ly6-G and CD45. Graph shows mean percentages of GFP^+^CD45^int^Ly6-G^high^ cells or median fluorescence of GFP in CD45^int^Ly6-G^high^ cells +/− SEM, respectively. (**B**–**E**) Mice were i.v. infected with 1.5 × 10^5^ CFU live *C. albicans* blastoconidia and treated with vehicle or 10 mg/kg ONX 0914 (s.c.) on the day of infection. Graphs show median fluorescence +/− SEM of CD11b expression on (**B**) kidney or (**D**) brain infiltrating CD45^int^Ly6-G^high^ neutrophils and MHC class II expression on (**C**) kidney infiltrating MHC-II^+^F4/80^+^CD11b^+^ macrophages or (**E**) brain infiltrating CD45^high^ CD11b^+^ myeloid cells, respectively. Data were pooled from two independent experiments (n = 7–10 mice per group). Data were analyzed by students t test with *p < 0.05 and ***p < 0.001. (**F**) NADPH oxidase activity as determined by DHR test. Isolated human neutrophils were treated with DMSO or 200 nM ONX 0914 *in vitro* for one hour and stimulated with 140 nM PMA for 30 min. Graph shows result from one experiment representative for three different blood donors.

Increased renal fungal burden during systemic *C. albicans* infection suggests impaired fungal control by innate immune cells. Especially neutrophils represent key innate immune effector cells that play a crucial role in phagocytosis and killing of *C. albicans*^34^. To assess the ability of neutrophils to kill *C. albicans*, mice were infected with a strain of GFP-positive *C. albicans* and the GFP signal among kidney neutrophils was measured. We found no difference in the percentage of GFP^+^ neutrophils between ONX 0914 treated and control mice (Fig. 8A) indicating that the phagocytic activity of neutrophils is not affected by LMP7 inhibition. However, there was a slightly (not quite significantly) higher median fluorescence of GFP within neutrophils of ONX 0914 treated mice which suggests that killing efficiency of neutrophils might be reduced by LMP7 inhibition (Fig. 8A). Moreover, we observed that neutrophils of ONX 0914 treated mice in the kidney as well as in the brain expressed reduced levels of the activation marker CD11b (Fig. 8B,D). Additionally, infiltrating macrophages in the kidney and myeloid cells of the brain expressed significantly lower levels of MHC class II supporting the hypothesis that LMP7 inhibition interferes with proper activation of innate immune cells in response to *C. albicans* (Fig. 8C,E). However, ONX 0914 treatment did not alter the capacity of isolated human neutrophils to produce reactive oxygen species (ROS) *in vitro* (Fig. 8F).

## Discussion

Besides its important role in MHC class I antigen processing, the immunoproteasome was found to be involved in proinflammatory immune responses and T helper cell differentiation^9^,^11^ while immunoproteasome inhibition ameliorated the outcome of several autoimmune diseases^9^,^10^,^11^,^12^,^13^,^14^. However, T helper cells do not only exert pathogenic functions in autoimmune disorders but also play a central role in host defence against for example fungal infections. In this study, we have investigated whether LMP7 inhibition would also affect T helper cell differentiation in response to *C. albicans,* a strong inducer of Th1 and Th17 cells^17^,^18^,^19^. Indeed, we found a reduced production of the Th1- and Th17-derived cytokines IL-17A and IFN-γ by ONX 0914 treated human PBMCs and murine splenocytes *in vitro* stimulated with heat-killed *C. albicans* (Fig. 1A,B). IL-17A and IFN-γ might also be produced by other cell types present in bulk splenocytes or PBMCs, however, treatment of CD4^+^ T cells was sufficient to reduce their release into the supernatant although not quite significantly for IFN-γ (Supplementary Fig. S2B). Moreover, IL-17A production was strongly dependent on MHC-II antigen presentation, indicating that LMP7 inhibits release of IL-17A by T helper cells (Supplementary Fig. S2C). IFN-γ and IL-17A production was also strongly reduced when only the ‘antigen presenting cells’-containing fraction (splenocytes - CD4^+^ T cells) was treated with ONX 0914 (Supplementary Fig. S2A), suggesting that immunoproteasome inhibition affects T helper cells as well as antigen presenting innate immune cells. Corroboratively, LMP7 inhibition *in vivo* led to a reduced generation of IL-17A- and IFN-γ- producing cells in mice systemically infected with *C. albicans* (Fig. 2) supporting the idea that LMP7 inhibition interferes with the differentiation of Th1 and Th17 cells.

ONX 0914 treated mice displayed a higher susceptibility to systemic candidiasis compared to vehicle treated control mice which, unexpectedly, manifested at very early stages of infection. We observed a more pronounced weight loss, an impaired survival, and a higher fungal burden of the kidney and the brain (Figs 3B,C and 4A,B). Interestingly, mice lacking immunoproteasome subunits LMP2, MECL-1, or LMP7 showed no difference in weight loss (Fig. 3A) or survival (data not shown) compared to WT mice. The difference between LMP7^−/−^ and ONX 0914 treated mice might be explained by structural changes in the immunoproteasome when LMP7 is absent and replaced by its constitutive counterpart β5 which maintains the chymotrypsin-like activity in immunoproteasomes of LMP7^−/−^ cells^35^. In contrast, when LMP7 is chemically inhibited with ONX 0914, the LMP7-dependent chymotrypsin-like activity of the immunoproteasome is irreversibly blocked. However, the influence of LMP7 inhibition became apparent far too early for impaired T helper cell differentiation being the reason for the increased susceptibility of ONX 0914 treated mice. In fact, adaptive immunity does not play a prominent role in combatting disseminated *candidiasis*. Instead, resistance to systemic *C. albicans* infection is mainly mediated by innate immune cells indicating that immunoproteasome inhibition has a more versatile effect on the anti-*C. albicans* immunity^25^,^30^,^36^.

Neutrophils are key innate immune effector cells that play a crucial role in phagocytosis and killing of *C. albicans*^20^,^34^,^37^. Therefore, it was surprising to observe strongly increased neutrophil numbers (Fig. 5) and higher fungal burden in the brains and kidneys of ONX 0914 treated mice (Fig. 4A,B) at the same time. Excessive neutrophil accumulation is most probably a compensatory event to fight increased renal fungal burden which might in turn result from impaired candidacidal activity of neutrophils and/or other innate immune cells as observed by others^38^. Upon PMA stimulation of purified PMNs *in vitro*, we observed no effect of LMP7 inhibition on the production of ROS (Fig. 8F), representing an important killing mechanism of neutrophils. However, killing of *C. albicans* by neutrophils occurs intracellularly and extracellularly as well as through oxidative and non-oxidative mechanisms. Hence, LMP7 might affect one or several non-oxidative killing mechanisms like for example the ability of neutrophils to phagocytose *C. albicans* or to form extracellular traps by releasing decondensed chromatin fibres decorated with antifungal proteins^15^,^39^. Moreover, LMP7 inhibition might influence neutrophil activity in an indirect manner. In fact, DCs which are strongly reduced in the brains and kidneys of ONX 0914 treated mice (Fig. 5C,D), were shown to be essential to sustain the anti-microbial activity of neutrophils via the induction of natural killer (NK) cells to produce GM-CSF^38^. Correspondingly, brain and kidney neutrophils of ONX 0914 treated mice displayed reduced expression of the activation marker CD11b which is part of the complement receptor CR3 and important for phagocytosis of *C. albicans* (Fig. 8B,D). Recently, Whitney *et al*. used GFP^+^ *C. albicans* to assess killing capacity of neutrophils *in vivo.* They observed a greater frequency of GFP^+^ neutrophils in CD11cΔSyk mice than in controls and concluded that kidney neutrophils from the former strain are impaired in their ability to destroy the fungus^38^. We found no difference in the percentage of GFP^+^ *C. albicans* containing neutrophils (Fig. 8A). However, this could mean that either uptake and killing is not affected by LMP7 inhibition or that neutrophils of ONX 0914 treated mice have reduced phagocytic activity. However, we noticed slightly enhanced fluorescence of GFP (reflecting the number of viable GFP^+^ *C. albicans)* within GFP^+^ neutrophils which might suggest reduced killing capacity *in vivo*. Additionally, we detected strongly decreased MHC class II expression on the surface of myeloid cells in the brain and macrophages in the kidney of ONX 0914 treated mice (Fig. 8C,E) suggesting that LMP7 inhibition interferes with a proper activation of innate immune cells and, consequently, probably also with an effective fungal control by these cells.

In order to characterize the influence of LMP7 inhibition on neutrophils in systemic candidiasis, we wanted to investigate whether increased neutrophil numbers result from an enhanced recruitment from peripheral blood. Interestingly, we observed elevated blood neutrophils in ONX 0914 treated mice particularly at later stages of disease (Fig. 6A). We found no difference in the percentage of neutrophils in the bone marrow between ONX 0914 treated and vehicle treated mice (Fig. 6B) suggesting that LMP7 inhibition has no impact on granulopoiesis but only leads to increased neutrophil recruitment to the site of infection or, possibly, to reduced neutrophil clearance in the periphery.

Interestingly, ONX 0914 treatment did only result in increased fungal burden in the kidney and the brain but not in the liver (Fig. 4). Hence, the question arises why control of *C. albicans* growth is only affected in the brain and in the kidney which are, also under “normal” circumstances, the most affected organs during systemic candidiasis^37^. Failure of fungal clearance might relate to organ-specific factors that impair neutrophil function, such as the high osmolarity and urea content of renal tubules, or, for example, to the induction of regulatory responses^33^,^40^. Indeed, hyphal forms of *C. albicans*, which are only found in the kidney (but not in the spleen or in the liver), are more resistant to killing by phagocytes than yeasts and too large to be ingested by neutrophils, which instead degranulate and release oxidative contents extracellularly thereby contributing to tissue damage^33^. Furthermore, hyphae induce a more anti-inflammatory profile than the yeast form, suggesting that morphogenetic changes can modulate the immune response to the advantage of the fungus. Conveniently, the PRR genes most highly upregulated in response to infection in the kidney were TLR2 and dectin-2, which is involved in the recognition of *C. albicans* hyphae^37^. Although TLR2 signaling can induce the production of proinflammatory mediators such as MIP-2, KC, TNF-α, IL-1β, and IFN-γ^37^,^41^, it can also induce IL-10 production and the expansion of Treg cells, suppressing immune responses to *C. albicans*^42^,^43^,^44^. Likewise, the anti-inflammatory milieu within the immune-privileged CNS might also contribute to reduced anti-*C. albicans* immunity within the brain^45^,^46^,^47^,^48^,^49^. Interfering with proinflammatory immune responses by LMP7 inhibition might further enhance the advantageous environment for fungal growth within these organs.

It has been demonstrated that disease severity in *C. albicans* infections correlates with kidney levels of many cytokines and chemokines like IL-6, KC (CXCL1), MIP-2 (CXCL2), TNF, and MCP-1 (CCL2)^30^. Interestingly, these cytokines and chemokines were strongly upregulated in ONX 0914 treated mice (Figs 6D,E and 7B). In particular, MacCallum *et al*. demonstrated significant correlation of early kidney KC (CXCL1) concentrations with subsequent histological kidney lesion parameters^30^. Hence, the development of tissue pathology in *C. albicans*-infected mice appears to depend on relative balances of cytokine or chemokine production and of neutrophil recruitment to the kidney supporting a direct exacerbating effect of LMP7 inhibition on the pathogenesis of disseminated candidiasis. Although neutrophils represent a central arm of the anti-*C. albicans* immunity they may also be harmful to the host by mediating immunopathology and tissue injury^31^. While sepsis is the major cause of death in systemic candidiasis, the extent of kidney damage in animals showing severe symptoms contributes to the overall pathology of the disease^28^,^30^. It has been recognized for many years that the disease processes in *C. albicans*-infected kidneys lead to heavy host leukocyte infiltrates and microabscess formation which in turn result in tissue damage^30^. Hence, excessive infiltration of monocytes and neutrophils into the brain and the kidney of ONX 0914 treated mice (Fig. 5C,D) might lead to meningoencephalitis and kidney injury, respectively^29^,^31^,^33^,^50^. Indeed, at least in some experiments, we noticed that kidneys of ONX 0914 treated mice had severe swelling and pallor in gross pathology and that these mice display altered behaviour as manifested by movement disorders and neurological abnormalities (data not shown). Moreover, although the expression of KIM-1, a marker of tubular epithelial damage currently used for diagnosis of acute kidney injury in humans^51^, was not changed (Fig. 7C), we measured significantly elevated TNF-α and IL-6 (Fig. 7B) as well as urea and creatinine (Fig. 7A) levels in the serum of ONX 0914 treated mice. This suggests that LMP7 inhibition contributes to both death-associated pathologies, sepsis as well as renal failure, respectively.

Taken together, it appears that LMP7 inhibition leads to reduced Th1 and Th17 cell differentiation in response to *C. albicans* but also affects the innate immunity resulting in higher susceptibility of ONX 0914 treated mice to systemic candidiasis. Hence, the question arises whether immunoproteasome inhibitors can in general be applied in the therapy of autoimmunity if they interfere with host defence against pathogens like fungi. Proteasome inhibitors are already used in humans e.g. for the treatment of multiple myeloma and, importantly, except for an increased incidence of varicella herpes zoster in bortezomib treated patients, there is no evidence for higher susceptibility to fungal infections^52^,^53^. Moreover, during infection with WT vaccinia virus (VV-WR) or lymphocytic choriomeningitis virus (LCMV-WE), we observed no influence on the immune system’s capability to clear the virus in mice treated with ONX 0914^9^. Nevertheless, it would be worthwhile to investigate the influence of LMP7 inhibition for example on oropharyngeal candidiasis (OPC) where host resistance is strictly dependent on T helper cells^23^. However, since even systemic candidiasis can be easily controlled with standard fungicide therapy (Supplementary Fig. S3), our data do not discourage the development and testing of LMP7-selective inhibitors as therapeutics against autoimmune diseases and inflammatory disorders.

To our knowledge this is the first time that ONX 0914 treatment was demonstrated to affect the innate immunity. An underlying mechanism could be reduced IL-23 secretion by dendritic cells and tissue-resident macrophages as observed for human PBMCs^9^,^13^. This would, for example, affect IL-23R^+^ IL-17-producing immune cells like γδT cells, iNKT cells, innate lymphoid cells (ILCs), or nTH17 cells which are implicated in host defence against *C. albicans*^54^,^55^,^56^. Moreover, Whitney *et al*. recently demonstrated that IL-23 produced by DCs is essential to induce GM-CSF production in NK cells which in turn sustains anti-microbial activity of neutrophils, the main fungicidal effectors^38^,^57^. Hence, it is of great interest to further investigate the role of the immunoproteasome in innate immunity and to determine the underlying mechanism of its influence on host defence against *C. albicans* and other pathogens.

## Material and Methods

### Mice

C57BL/6 mice (H-2^b^) were originally purchased from Charles River, Germany. MECL-1^58^, LMP2^59^, and LMP7 gene-targeted mice^60^ were provided by John Monaco (University of Cincinnati, Cincinnati, OH). Mice were kept in a specific pathogen-free facility and used at 8–10 weeks of age. Animal experiments were approved by the Review Board of Governmental Presidium Freiburg of the State of Baden-Württemberg. All methods were carried out in accordance with the approved guidelines.

### Immunoproteasome inhibition

For *in vitro* experiments, the LMP7-selective inhibitor ONX 0914 (Onyx Pharmaceuticals) was dissolved at a concentration of 10 mM in DMSO and stored at −80 °C. For *in vivo* proteasome inhibition, ONX 0914 was formulated in an aqueous solution of 10% (w/v) sulfobutylether-β-cyclodextrin and 10 mM sodium citrate (pH 6) referred to as vehicle and administered to mice as an s.c. bolus dose of 10 mg/kg.

### Systemic infection with *C. albicans*

The *Candida albicans* laboratory strain SC5314 (kind gift from Prof. Joachim Morschhäuser, Institute for Molecular Infection Biology, University of Würzburg) or *C. albicans* strain CAI4-pACT1 GFP (kind gift from Prof. Salome LeibundGut, Institute of Microbiology, ETH Zürich) was grown on YPD plates at 30 °C. A single colony of *C. albicans* was grown for 18 h at 30 °C in YPD media and yeast cells were harvested by centrifugation, washed with PBS, and counted with a hemocytometer. Mice were injected i.v. with blastoconidia in PBS and their health status was monitored daily. Animals that became immobile or otherwise showed signs of severe illness were humanely terminated and recorded with 30% weight loss and as dying on the following day. For *in vitro* experiments, *C. albicans* yeast and hyphae were heat-killed for 1 h at 100 °C. To generate pseudohyphae, blastoconidia were grown at 37 °C and 6% CO_2_ in serum-free RPMI medium overnight.

### Fungal burden

To assess the tissue outgrowth of *C. albicans*, kidneys, brains, and livers of the sacrificed animals were removed aseptically, weighed, and homogenized in sterile distilled water using a tissue homogenizer. The number of viable *C. albicans* cells in the tissues was determined by plating serial dilutions on YPD plates. The colonies were counted after 24 h of incubation at 30 °C, and the fungal burden was expressed as log_10_ CFU/g tissue.

### Antifungal therapy

Amphotericin B was purchased as Fungizone^TM^ (Medicopharm AG, Germany). Amphotericin B as raw material (50 mg) was dispersed in 10 ml sterile distilled water and aliquots were stored at −20 °C. Amphotericin B stock solution (5 mg/ml) was diluted with 5% glucose (pH > 4.2) and administered intraperitoneally as a single bolus of 10 mg/kg every day.

### *Ex vivo* cytokine production by primed splenocytes

To assess *in vivo* T helper cell differentiation, ~5 × 10^6^ primed spleen cells from *C. albicans* infected mice were *in vitro* restimulated with heat-killed *C. albicans* yeast (~10^6^ cells/mL) and cultured for 48 h at 37 °C. Culture supernatants were analyzed for IL-17A and IFN-γ by ELISA according to the manufacturer´s protocol (eBioscience).

### *In vitro* T helper cell differentiation

Human PBMCs from healthy volunteers (2.5 × 10^5^/well) or bulk splenocytes of naive C57BL/6 (2 × 10^5^/well) were pulsed for 2 h with 200 nM ONX 0914 at 37 °C, washed and cultured in the presence of heat-killed *C. albicans* yeast or hyphae (~1 × 10^6^ cells/ml) in IMDM at 37 °C. Cytokines in the supernatant were determined by ELISA according to the manufacturer’s protocol (eBioscience). In some experiments (Supplementary Fig. S2A, 2B), splenocytes were magnetically sorted (MACS) for CD4^+^ T cells using a negative sorting approach. Either CD4^+^ T cells or the ‘antigen presenting cell’-containing cell fraction (splenocytes - CD4^+^ T cells) were pulsed with ONX 0914 before combining both fractions and stimulating with *C. albicans*.

### Quantitative real-time RT-PCR

Real-time RT-PCR was used to quantify cytokine expression levels in mouse kidney as previously described^13^ with slight modifications. RNA from mouse kidney was extracted with the RNeasy mini plus kit of Qiagen. One μg of total RNA was reverse transcribed using oligonucleotide (dT) primers (see Table 1) and the reverse transcription system (Promega). Gene expression was normalized to glycerolaldehyd-3-phosphate-dehydrogenase (GAPDH) as a reference gene and results for all mice are expressed relative to one naive uninfected mouse (fold induction over naive).

### Isolation of leukocytes from kidney

Mice were sacrificed and leukocytes from the kidney were isolated by enzymatic digestion of tissues with collagenase D (0.2 mg/ml) and DNase I (0.2 mg/ml) followed by Percoll^®^ density gradient (70%/30%) centrifugation. Cells were collected from the interphase and analyzed for surface marker expression by flow cytometry.

### Isolation of leukocytes from brain

Mice were sacrificed and perfused with cold PBS to minimize contamination of brain and spinal cord with blood-derived leukocytes from peripheral blood. Leukocytes from CNS were isolated by enzymatic digestion of brains followed by Percoll^®^ density gradient centrifugation as previously described^13^.

### Flow cytometry

Flow cytometry was performed as previously described^13^. Abs to Ly6-G (RB6-8C5), CD45 (30-F11), IL-17A (eBio17B7), F4/80 (BM8), CD11b (M1/70), CD11c (HL3), MHC-II (AF6-120.1), were obtained from BD Biosciences or eBioscience. Anti-IFN-γ antibody (AN18) was kindly provided by Dr. Michael Basler. Cells were acquired with the use of the BD Accuri^TM^ C6 flow cytometer system and pregated on living cells according to FSC/SSC signal.

### Bead-based cytokine assay

Serum levels of TNF-α, IL-6, and KC (CXCL1) were determined by multiplexed bead-based assays (Bio-Plex Pro Mouse Cytokine Assays, Bio-Rad Laboratories, Hercules, CA). Samples were prepared according to the manufacturer’s instructions and were analyzed on a FACSCanto II flow cytometer (BD Immunocytometry Systems, Heidelberg, Germany). Absolute cytokine concentrations were calculated based on the mean fluorescence intensity of cytokine standards with a 4-parameter logistic curve model. The sensitivity of the assays was 1.4 pg/ml for TNF-α, 0.2 pg/ml IL-6, and 0.3 pg/ml for KC.

### Photometric assays

Serum creatinine and urea were measured photometrically using an automated clinical chemistry analyser (*ADVIA 1800*, Siemens) at the medical laboratory Dr. Brunner in Konstanz.

### Isolation of polymorphonuclear cells (PMNs) from human peripheral blood

Neutrophils were isolated using a density gradient centrifugation method. In brief, 1 part of 1× HBSS diluted blood (without Ca^2+^/Mg^2+^) was carefully underlayed by Ficoll Paque^TM^ Plus (δ = 1.077 g/ml) and centrifuged at 400 × g, 20 °C for 40 min without brake. Supernatant and mononuclear cell fraction were removed and the pellet was washed in HBSS. Erythrocytes were lysed with H_2_O for 20 s and PMNs were washed and resuspended in HBSS.

### Isolation of peripheral blood mononuclear cells (PBMCs) from human blood

Human PBMCs were isolated from whole blood with the help of BD Vacutainer^®^ CPT^TM^ and cultured in RPMI medium.

### NADPH oxidase activity assay

PMNs (5.0 × 10^6^ cells/ml) that were previously treated with DMSO or 200 nM ONX 0914 for 1 h at 37 °C were activated or not with 140 nM PMA for 30 min at 37 °C, respectively. After stimulation, the cells were incubated with 50 μM DHR for 30 min, washed once, and resuspended in HBSS. The fluorescence of gated neutrophils was detected in FL1 using the BD Accuri^TM^ C6 flow cytometer.

### Statistical analysis

The statistical significance was determined using the students t test, two-way ANOVA or non-parametric Mann-Whitney test with two-tailed P value. All statistical analyses were performed using GraphPad Prism Software (version 4.03) (GraphPad, San Diego, CA). Statistical significance was achieved when p < 0.05. If not indicated otherwise, differences are not significant.

## Additional Information

**How to cite this article**: Mundt, S. *et al*. Inhibiting the immunoproteasome exacerbates the pathogenesis of systemic *Candida albicans* infection in mice. *Sci. Rep.* **6**, 19434; doi: 10.1038/srep19434 (2016).

## Supplementary Material

Supplementary Information

## Figures and Tables

**Table 1:** PCR primers used in this study.

Target gene	Fwd Primer	Rev primer
MIP-2α	5′ -AAGTTTGCCTTGACCCTGAA-3′	5′ -AGGCACATCAGGTACGATCC-3′
KC	5′ -TGGCTGGGATTCACCTCAAG-3′	5′ -CCGTTACTTGGGGACACCTT-3′
MIP-1α	5′ -GTAGCCACATCGAGGGACTC-3′	5′ -GATGGGGGTTGAGGAACGTG-3′
MCP-1	5′ -TCAGCCAGATGCAGTTAACG-3′	5′ -GTTGTAGGTTCTGATCTCATTTGG-3′
KIM-1	5′ -ACATTCTCCGTAAATGGGCTT-3′	5′ -CTGCTGTGAAGGAGACCCTG-3′
