# Supplementary material for: Inhibiting the immunoproteasome exacerbates the pathogenesis of systemic Candida albicans infection in mice
Source: Sci Rep. 2016 Jan 18;6:19434. doi: 10.1038/srep19434 (PMC4726078; doi:10.1038/srep19434)
Supplement: Supplementary Information [file srep19434-s1.doc]

**Supplementary data for**

**Inhibiting the Immunoproteasome Exacerbates the Pathogenesis of Systemic *Candida albicans* Infection in Mice**

**Sarah Mundt, Michael Basler, Stefanie Buerger, Harald Engler, Marcus Groettrup**

**Scientific Reports**

**
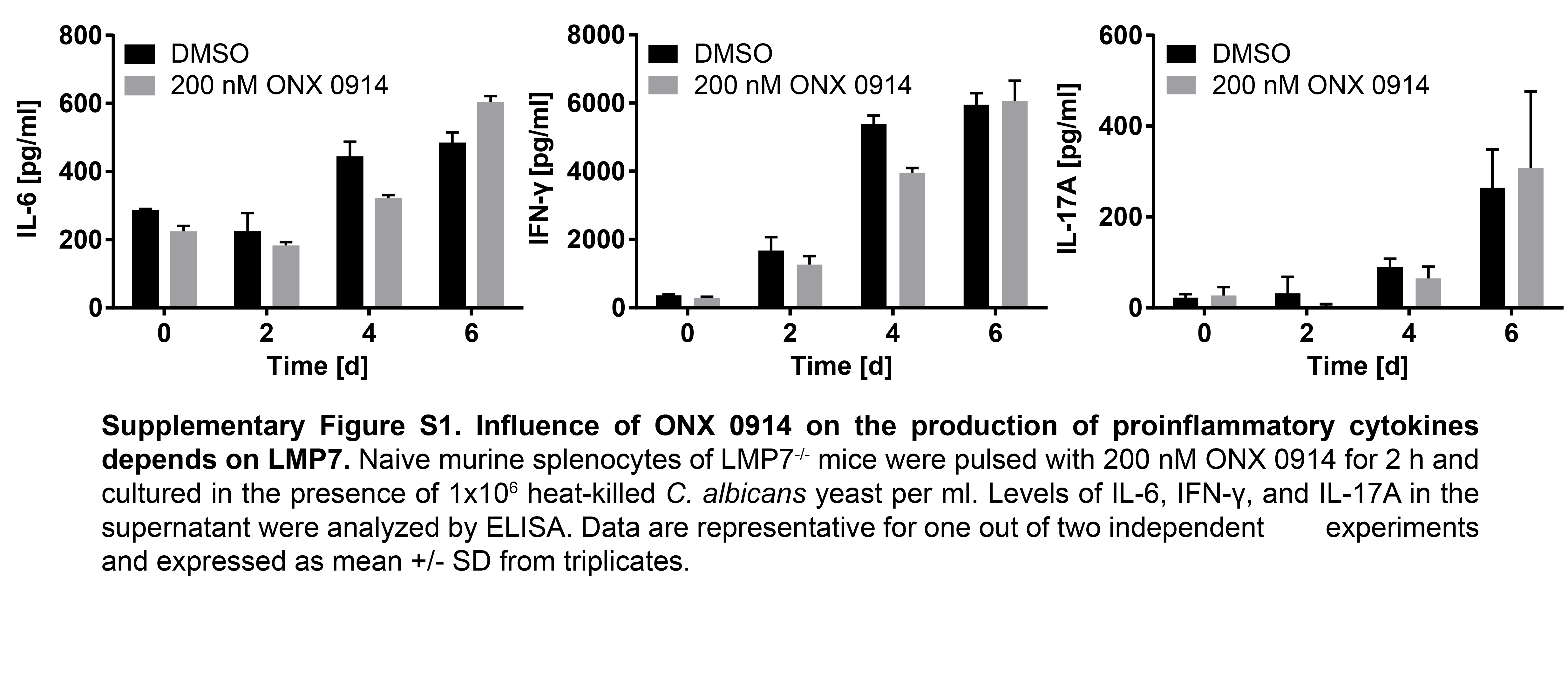
**

**Supplementary Figure S1. Influence of ONX 0914 on the production of proinflammatory cytokines depends on LMP7.** Naive murine splenocytes of LMP^7-/-^ mice were pulsed with 200 nM ONX 0914 for 2 h and cultured in the presence of 1x10^6^ heat-killed *C. albicans* yeast per ml. Levels of IL-6, IFN-γ, and IL-17A in the supernatant were analyzed by ELISA. Data are representative for one out of two independent experiments and expressed as mean +/- SD from triplicates.


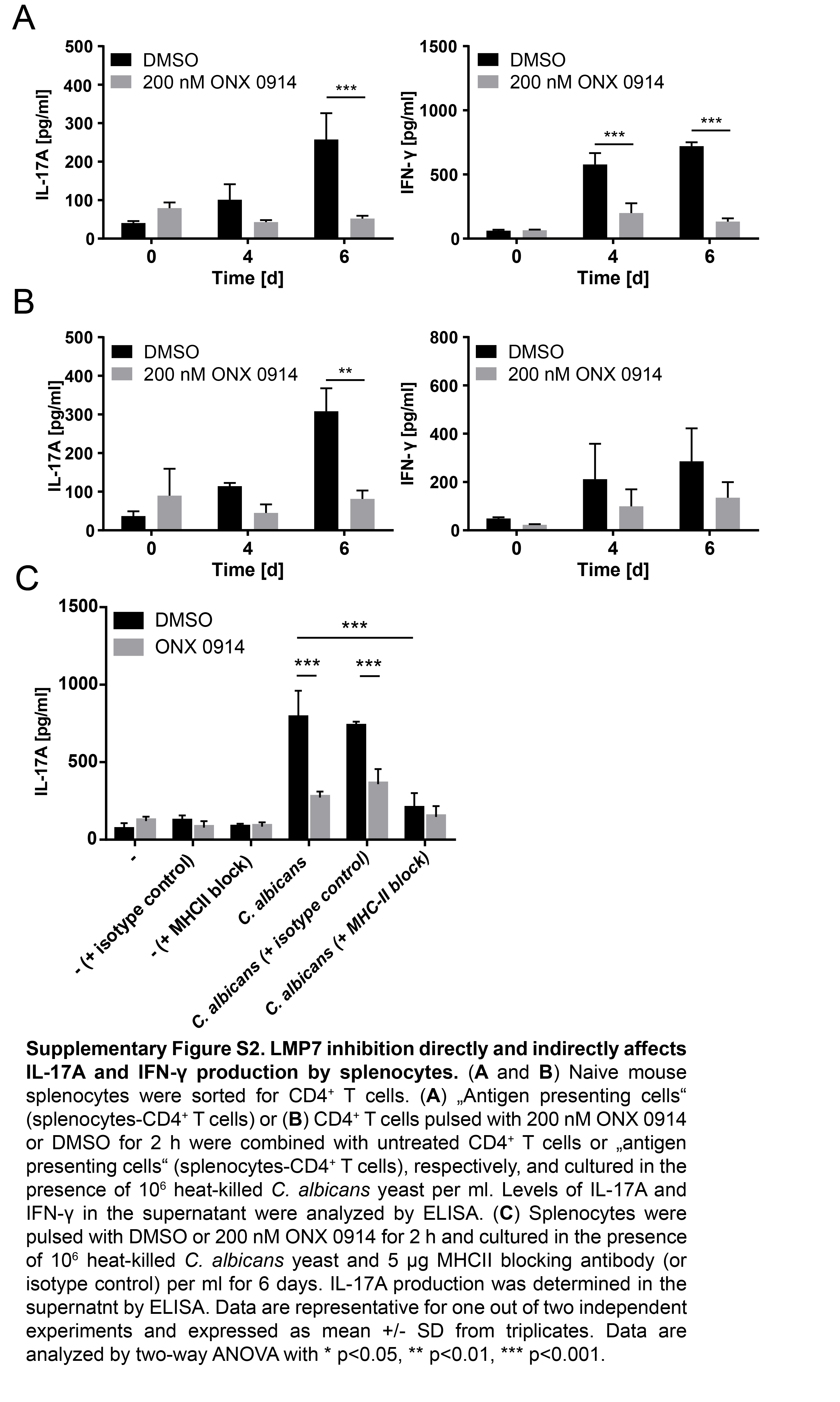


**Supplementary Figure S2. LMP7 inhibition directly and indirectly affects IL-17A and IFN-γ production by splenocytes.** (**A** and **B**) Naive mouse splenocytes were sorted for CD4^+^ T cells. (**A**) „Antigen presenting cells“ (splenocytes-CD4^+^ T cells) or (**B**) CD4^+^ T cells pulsed with 200 nM ONX 0914 or DMSO for 2 h were combined with untreated CD4^+^ T cells or „antigen presenting cells“ (splenocytes-CD4^+^ T cells), respectively, and cultured in the presence of 10^6^ heat-killed *C. albicans* yeast per ml. Levels of IL-17A and IFN-γ in the supernatant were analyzed by ELISA. (**C**) Splenocytes were pulsed with DMSO or 200 nM ONX 0914 for 2 h and cultured in the presence of 10^6^ heat-killed *C. albicans* yeast (“-“: not stimulated with *C. albicans*) and 5 μg MHC-II blocking antibody (or isotype control) per ml for 6 days. IL-17A production was determined in the supernatant by ELISA. Data are representative for one out of two independent experiments and expressed as mean +/- SD from triplicates. Data are analyzed by two-way ANOVA with * p<0.05, ** p<0.01, *** p<0.001.


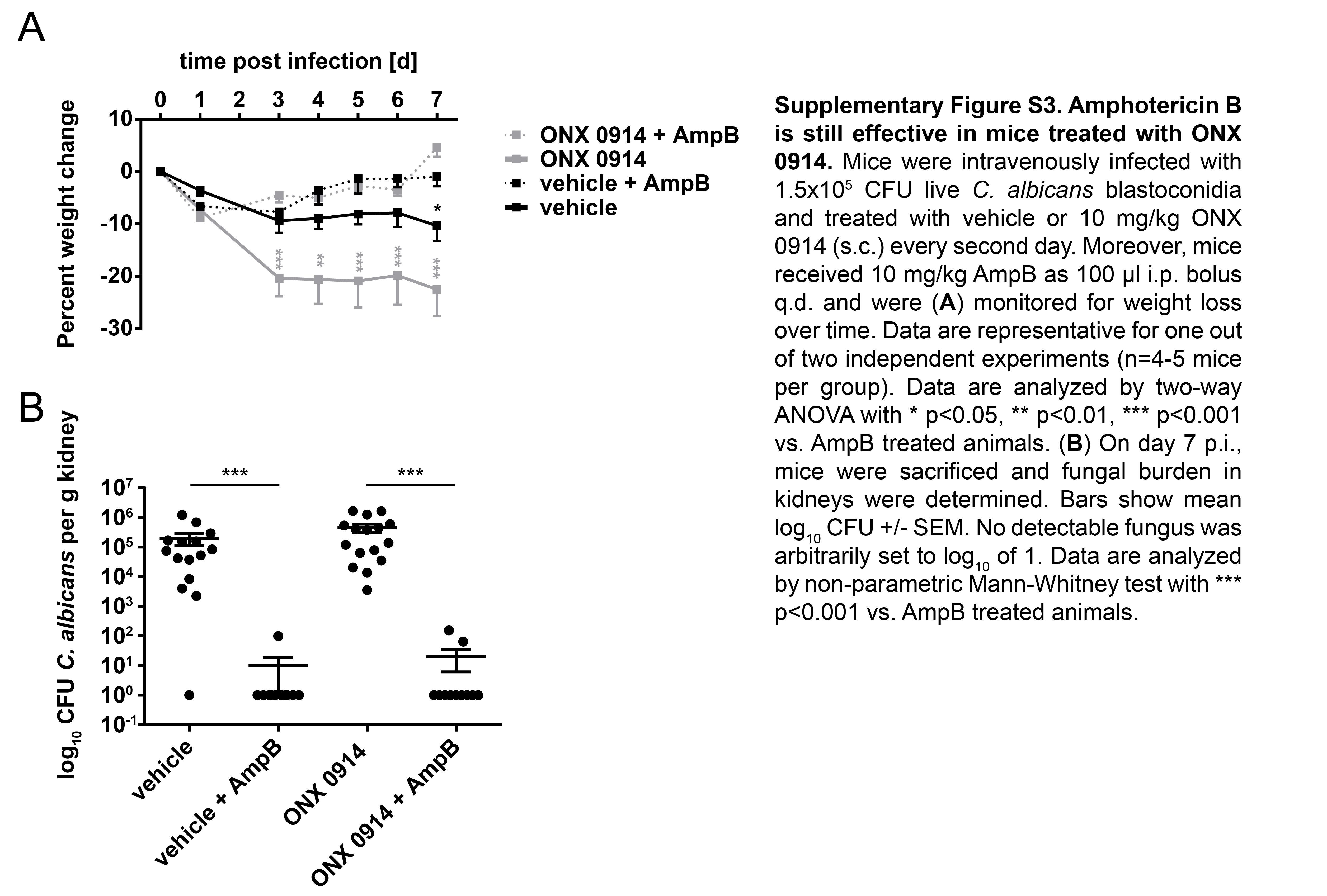


**Supplementary Figure S3. Amphotericin B is still effective in mice treated with ONX 0914.** Mice were intravenously infected with 1.5x10^5^ CFU live *C. albicans* blastoconidia and treated with vehicle or 10 mg/kg ONX 0914 (s.c.) every second day. Moreover, mice received 10 mg/kg AmpB as 100 µl i.p. bolus q.d. and were (**A**) monitored for weight loss over time. Data are representative for one out of two independent experiments (n= 4-5 mice per group). Data are analyzed by two-way ANOVA with * p<0.05, ** p<0.01, *** p<0.001 vs. AmpB treated animals. (**B**) On day 7 p.i., mice were sacrificed and fungal burden in kidneys were determined. Bars show mean log10 CFU +/- SEM. No detectable fungus was arbitrarily set to log10 of 1. Data are analyzed by non-parametric Mann-Whitney test with *** p<0.001 vs. AmpB treated animals.
